# Prenatal and Infant Reports and Child Protection Involvement: A Longitudinal Cohort Study

**DOI:** 10.1177/10775595251381279

**Published:** 2025-09-19

**Authors:** Miriam J. Maclean, Fernando Lima, Stephanie Taplin, Rhonda Marriott, Jacynta Krakouer, Melissa O’Donnell

**Affiliations:** 1 Australian Centre for Child Protection, 613331 University of South Australia, Adelaide, SA, Australia; 2 Telethon Kids Institute, Nedlands, WA, Australia; 3 School of Public Health, 110561 Faculty of Health, University of Technology Sydney, Sydney, NSW, Australia; 4 Ngangk Yira Institute for Change, 5673 Murdoch University, Murdoch, WA, Australia

**Keywords:** infants, child protective services, foster care

## Abstract

In Australia, infants have the highest rate of child protection involvement. Many jurisdictions in Australia and internationally have introduced policies for prenatal planning and support, however little is known about outcomes of infants reported prenatally. This study is the first to use cross-jurisdictional, individual-record data to examine child protection pathways associated with prenatal and infant reports. Australian Institute of Health and Welfare data covering 2012–2018 was used. Cox regression analyses examined factors associated with removal into out-of-home care and reunification. Removals were significantly more likely for children with prenatal reports (HR = 2.29, 95% CI: 2.17–2.41). Earlier-in-pregnancy reports were not associated with reduced removals. There was significant variation across jurisdictions in removals and reunifications. Aboriginal children were significantly more likely to have prenatal reports and removals and less likely to be reunified. Examining the effectiveness and potential improvement of prenatal interventions and support could increase children’s opportunity to safely remain at home.

## Introduction

The first years of life are a crucial stage of child development, which can be adversely affected by maltreatment (Cicchetti, 2016). Australian and international research indicates infants (aged 0–1 year) are particularly vulnerable due to their dependency and developmental stage, with the highest rates of maltreatment (Australian Institute of Health and Welfare, 2022; Eastman et al., 2016), and increasing rates of entry to out-of-home care (OoHC) (Gilbert et al., 2012). Given the vulnerability of infants, and recognition of the importance of early intervention in health and social policy there is increasing focus on this youngest group of children (Juhasz, 2020) and child protection services in many jurisdictions across Australia and internationally have introduced prenatal reporting. The aim of prenatal reporting is to identify the need for support during pregnancy to either prevent the need for OoHC or identify where removal of an infant may be considered necessary for their safety (Taplin, 2017).

OoHC is considered an intervention of last resort. Removing an infant from their family is traumatic, and can affect breastfeeding, bonding (Blythe et al., 2022) and cultural connection (Trew et al., 2022). The time between a prenatal report and birth provides a unique opportunity for early intervention prior to removal being an option, with early-pregnancy reports allowing more time for interventions to potentially be implemented and take effect.

Using this opportunity optimally to reduce risk should be prioritised. There is debate and disquiet regarding the removal of infants shortly after birth in the UK, USA, Canada, Australia and Europe both overall and due to the impact on First Nations families (Broadhurst et al., 2022). Internationally, First Nations children are disproportionately represented in OoHC (Brownell et al., 2015; Office of the Children’s Commissioner, 2020) with the OoHC rate among Aboriginal and Torres Strait Islander infants in Australia almost 10 times that of non-Aboriginal infants (O'Donnell et al., 2019). We respectfully use the terms ‘Aboriginal' and ‘Aboriginal and Torres Strait Islander' interchangeably throughout this article to refer to First Nations Peoples in Australia. The Australian Government’s Closing the Gap strategy includes a target of reducing overrepresentation of Aboriginal children in the child protection system (Commonwealth of Australia, 2020). A detailed understanding of where in the child protection pathways this overrepresentation occurs and can be addressed within the child protection system is needed to determine future action.

Introduced initially in New South Wales (NSW) in 1998 (Swain, 2014), prenatal reporting has been introduced in several Australian States and provides a pathway for pre-birth child protection involvement. As Australian legislation generally treats a child as a legally distinct person from birth onwards, during pregnancy reports allege anticipated harm to an infant post-birth. Investigations which conclude there is reasonable cause to believe a child has been or is likely to be abused, neglected or otherwise harmed post-birth result in a substantiation of maltreatment (2). As this definition includes likelihood of harm, prenatal substantiations may take into account factors such as a history of sexual offences or maltreatment of the unborn child’s older siblings. Exposure to domestic violence is classed as a form of emotional abuse.

Prenatal reporting aims to increase support to families during pregnancy and post-birth, yet few studies have examined prenatal reporting outcomes. A study of 117 mothers reported prenatally in the Australian Capital Territory (ACT) in 2013 found that multiple prenatal reports were associated with increased likelihood of OoHC in early infancy, while timing (stage of pregnancy) of the prenatal report was not (Taplin, 2017). Just over half the women in the study had their first report occurring within the last three months of pregnancy. A NSW study of newborn infants entering OoHC from 2006 to 2014, the majority of whom had been the subject of a prenatal report, found only 6.6% of infants were reunified (Marsh et al., 2017). This suggests that for most families in this situation, removals may be long-term or permanent, despite the fact that reunification is considered a policy priority in Australia (Amos et al., 2022). It is unclear in the study by Marsh and colleagues (2017) whether reunification was being attempted unsuccessfully or whether the children remained continuously in OoHC. In an English study over the same time period, a higher proportion of newborn and older infant removals returned to parental care, with 20% of newborns successfully reunified and a further 5% reunified but then re-entering OoHC (Pearson et al., 2020). Understanding both the frequency of child protection outcomes such as entry to OoHC and reunification attempts, and the factors associated with these outcomes among children who are the subject of prenatal and infant reports is vital to inform policy and interventions.

To date, important knowledge has been gleaned about prenatal reporting using relatively small samples from case file extracts from single states (Meiksans et al., 2021; Taplin, 2017), or aggregated state level data (O’Donnell et al., 2023). A recent study (O’Donnell et al., 2023) found marked variation in rates of reports and infant OoHC entry across Australia; jurisdictions with prenatal reporting had the highest removal rates in the week after birth, however without individual-record data the study could not ascertain whether the infants removed had been the subject of prenatal notifications.

As each Australian state/territory has its own child protection jurisdiction, with different policies and practices regarding perinatal reporting and child protection processes, there is an opportunity to explore these differences via large state-based standardised datasets. The current study utilises longitudinal data from child protection populations across multiple Australian states and territories to understand the use and outcomes of prenatal reporting, including the following aims:

(i) identify timing of reports
(ii) examine characteristics associated with prenatal and infant reports
(iii) describe trajectories of children with prenatal or infant reports through the child protection system, including the likelihood of removal, and reunification.
(iv) extend knowledge about similarities and differences across jurisdictions regarding the use and sequalae of prenatal and infant reports.

## Method

### Data Source

The Australian Institute of Health and Welfare (AIHW) National Child Protection Minimum Dataset was utilised covering the period from the 1st July 2012 to the 31st August 2018. Australia has eight States and Territories: Victoria (Vic), New South Wales (NSW), Western Australia (WA), Queensland (Qld), South Australia (SA), Northern Territory (NT), Tasmania (Tas), and the Australian Capital Territory (ACT). Across Australia, the eight state/territory child protection departments collect individual child-level data which is provided to AIHW according to national agreed definitions and specifications (except NSW who provide aggregate data and are therefore excluded from the current study). For Queensland (Qld), data is available from 2014–15 onwards and may not be comparable to data from previous years or match Queensland figures published elsewhere.

In most jurisdictions, prenatal reports can be made, however legislation and responses vary. Victoria (Vic) does not investigate and substantiate prenatal reports, and engagement with pre-birth processes is voluntary (Victorian Department of Families Fairness and Housing, 2023); consequently their prenatal report data cannot be collected for comparative purposes. Northern Territory (NT) had very few prenatal reports; these are not reported separately. All seven included jurisdictions provided data on infant reports, substantiations and OoHC, however reunification data was not available for Queensland.

For this paper, Vic, Western Australia (WA), Qld and NT have authorised reporting of individual state data, but South Australia (SA), Tasmania (Tas) and the Australian Capital Territory (ACT) have only authorised reporting of combined data from their three states/territories. To meet privacy requirements, due to the small number of prenatal reports for NT, prenatal data is aggregated along with ACT, SA and Tas, with only the postnatal results presented separately. As noted above, NSW was the one state that could not be included as individual record level data was not available.

### Setting

The study population includes seven of the eight states/territories of Australia. Vic is the most populous included at 6.5 million, but has the smallest proportional Aboriginal population (1.0%). Conversely, NT’s population is 232,605 but has proportionally the largest Aboriginal population (26.3%) (Australian Bureau of Statistics, 2021b, Australian Bureau of Statistics, 2021a).

### Study Population

The AIHW National Child Protection Minimum Dataset included data from the 1st July 2012 to the 31st August 2018. The study cohort included 50,196 children who were the subject of one or more child protection reports during pregnancy or infancy (birth to age 1). In all states except Qld, children born from 1st April 2013 onwards were included, to ensure the reports data from 1st July 2012 covered the duration of pregnancy. Qld reports data began in July 2014, so children born from April 2015 were included to allow for reports occurring during pregnancy. For all states, data (including births and child protection events) was available until 2018. Children (*n* = 838) reported prenatally with no birthdate subsequently recorded (none had an OoHC record) are included, but cannot be used in time to event analyses.

### Variables

Child protection variables included children’s number of reports, report timing (prenatal versus postnatal), time of prenatal report (1 month before birth or less, 1–3 months, 3–6 months, and 6–9 months), prenatal substantiations, primary maltreatment type for first prenatal substantiation, time to first OoHC entry from birth, and time to first reunification event from first OoHC entry. Jurisdictions vary substantially in thresholds for notifications and investigations and whether multiple contacts to child protection regarding one episode are recorded as one or multiple reports (see Child Protection Australia Appendix (Australian Institute of Health and Welfare, 2022), for further detail). This was addressed by including jurisdiction-stratified analyses and collapsing number of notifications into categories (1, 2–5, 6+). Analysis focussed primarily on counting children who experienced child protection events. Demographic information included sex, Aboriginal and Torres Strait Islander status (yes versus no/unknown), and state/territory.

### Ethics

Ethical approval was provided by the Australian Institute of Health and Welfare Ethics Committee (reference EO2019/3/1053).

### Analysis

In addition to descriptive statistics, logistic regression assessed demographic characteristics associated with having a prenatal report. Bivariate Cox regression analyses examined relationships between each predictor variable and the relative likelihood of entry to OoHC at any point in time over the first year of life (in days). Children were censored at the end of the follow-up period (31 August 2018). As a number of variables did not meet the proportional hazards assumption, time-stratified analyses were used to investigate associations over time. Stratified analyses by Aboriginal and Torres Strait Islander status and jurisdiction were conducted to assess whether findings varied between these groups. Bivariate Cox regressions assessed relationships between predictor variables and reunification.

## Results

### Characteristics of the Prenatal/Infant Cases

The study included 50,196 children who were the subject of at least 1 prenatal and/or infant child protection notification. Almost half were recorded as male (47.9%), 44.8% female and 7.3% not stated (sex was less likely to be recorded in prenatal notifications). A quarter of the children were Aboriginal and/or Torres Strait Islander (25.6%). Vic provided 43.4% (*N* = 21,793) of cases. ACT, SA, and Tas together comprised 22.1% (*N* = 11,104), Queensland 14.1% (*N* = 7,075), Western Australia 14.0% (*N* = 7,010) and NT 6.4% (*N* = 3,214).

#### Prenatally Reported Cases

Among the study children 9,279 (18.5%) had a prenatal report (Table 1). A third of these (32.4%) had a prenatal substantiation. The first substantiation for prenatal reports was most commonly neglect (60.5%), followed by emotional (18.6%) and physical abuse (17.9%). More than half prenatally-reported children were first reported at least 3 months before birth. Only 14.6% were first reported within 1 month before birth. Most (83.9%) had a single prenatal report, and 60.3% had no postnatal reports during the study. However, 18.6% had 1 postnatal report, 17.4% had 2–5, and 3.7% had 6 or more postnatal report.

**Table 1.** Characteristics Children First Reported Prenatally and Children First Reported Postnatally

Children reported prenatally		NT		Qld		Vic		WA		NT, SA, Tas, ACT		Total	
				*n* = 3666 (51.8%)				*n* = 3006 (42.9%)		*n* = 2607 (18.20%)		*n* = 9279 (18.5%)	
				*N*	%			*N*	%	*N*	%	*N*	%
Sex	Male	—	—	1,423	38.8%	—	—	1,221	40.6%	808	31.0%	3,452	37.2%
	Female	—	—	1,332	36.3%	—	—	1,120	37.3%	796	30.5%	3,248	35.0%
	Not recorded	—	—	911	24.8%	—	—	665	22.1%	1,003	38.5%	2,579	27.8%
Aboriginal and Torres Strait Islander	Yes	—	—	1,472	40.2%	—	—	1,441	47.9%	433	16.6%	3,346	36.1%
	No	—	—	1,588	43.3%	—	—	1,127	37.5%	914	35.1%	3,629	39.1%
	Not stated	—	—	606	16.5%	—	—	438	14.6%	1,260	48.3%	2,304	24.8%
Months from first prenatal report to birth	6–9	—	—	641	18.6%	—	—	273	9.3%	490	23.7%	1,404	16.6%
	3–6	—	—	1,456	42.3%	—	—	1,174	40.1%	804	38.8%	3,434	40.7%
	1–3	—	—	897	26.0%	—	—	983	33.6%	492	23.8%	2,372	28.1%
	Final month before birth	—	—	452	13.1%	—	—	495	16.9%	284	13.7%	1,231	14.6%
Prenatal reports (number)	1	—	—	3,209	87.5%	—	—	2,799	93.1%	1,774	68.0%	7,782	83.9%
	2–5	—	—	457	12.5%	—	—	207	6.9%	788	30.2%	1,452	15.6%
	6+	—	—	0	0.0%	—	—	0	0.0%	45	1.7%	45	0.5%
Postnatal reports (number)	0	—	—	2,506	68.4%	—	—	1,824	60.7%	1,267	48.6%	5,597	60.3%
	1	—	—	741	20.2%	—	—	614	20.4%	371	14.2%	1,726	18.6%
	2–5	—	—	<450	<12%	—	—	550	18.3%	645	24.7%	1,610	17.4%
	6+	—	—	<5	<0.5%	—	—	18	0.6%	324	12.4%	346	3.7%
Total reports (number)	1	—	—	2,236	61.0%	—	—	1,726	57.4%	1,017	39.0%	4,979	53.7%
	2-5	—	—	1,401	38.2%	—	—	1,232	41.0%	1,059	40.6%	3,692	39.8%
	6+	—	—	29	0.8%	—	—	48	1.6%	531	20.4%	608	6.6%
Any prenatal substantiation	No	—	—	2,056	56.1%	—	—	1,894	63.0%	2,326	89.2%	6,276	67.6%
	Yes	—	—	1,610	43.9%	—	—	1,112	37.0%	281	10.8%	3,003	32.4%
Primary type first substantiated (prenatal)	Physical	—	—	299	18.6%	—	—	221	19.9%	19	6.8%	539	17.9%
	Sexual	—	—	39	2.4%	—	—	<15	<2.0%	<5	<2.0%	55	1.8%
	Emotional	—	—	163	10.1%	—	—	284	25.5%	111	39.5%	558	18.6%
	Neglect	—	—	1,109	68.9%	—	—	582	52.3%	125	44.5%	1,816	60.5%
	Not stated	—	—	0	0.0%	—	—	<15	<2.0%	<25	<8.0%	35	1.2%

Children first reported postnatally		NT		Qld		Vic		WA		ACT, SA, Tas		Total	
		*n* = 3,207 (>99.5%)		*n* = 3,409 (48.2%)		*n* = 21,793 (100%)		*n* = 4004 (47.1%)		*n* = 8,504 (76.6%)		*n* = 40,917 (81.5%)	
Sex	Male	∼1,700	∼53.0%	1,728	50.7%	10,957	50.3%	1,842	46.0%	4,351	51.2%	20,581	50.3%
	Female	∼1,500	∼47.0%	1,610	47.2%	10,349	47.5%	1,829	45.7%	3,962	46.6%	19,253	47.1%
	Other/Not recorded	<5	<1%	71	2.1%	487	2.2%	333	8.3%	191	2.2%	1,083	2.6%
Aboriginal and Torres Strait Islander	Yes	2,634	82.1%	1,157	33.9%	2,506	11.5%	1,330	33.2%	1,897	22.3%	9,524	23.3%
	No	561	17.5%	1,729	50.7%	17,825	81.8%	1,757	43.9%	4,787	56.3%	26,659	65.2%
	Not stated	12	0.4%	523	15.3%	1,462	6.7%	917	22.9%	1,820	21.4%	4,734	11.6%
Postnatal reports	1	1,044	32.6%	2,437	71.5%	10,385	47.7%	2,191	54.7%	3,411	40.1%	19,468	47.6%
	2-5	1,458	45.5%	961	28.2%	9,306	42.7%	1,726	43.1%	3,780	44.4%	17,231	42.1%
	6+	705	22.0%	11	0.3%	2,102	9.6%	87	2.2%	1,313	15.4%	4,218	10.3%
Primary type first substantiated	Physical	245	19.3%	274	23.5%	1,952	18.2%	316	18.3%	633	24.9%	3,420	19.6%
	Sexual	<5	<0.5%	28	2.4%	645	6.0%	31	1.8%	71	2.8%	779	4.5%
	Emotional	467	36.8%	285	24.5%	7,037	65.8%	712	41.2%	798	31.4%	9,299	53.4%
	Neglect	554	43.6%	577	49.6%	1,000	9.3%	650	37.6%	1,022	40.2%	3,803	21.9%
	Not stated	<5	<0.5%	0	0.0%	64	0.6%	20	1.2%	20	0.8%	104	0.6%

*Notes.* Children with missing birth date included. Substantiation includes a substantiated risk of future harm, so substantiations (including prenatal substantiations) such as sexual abuse may consider factors such as abuse of older siblings or sexual offending history. Primary type of first substantiated maltreatment in prenatal reports section includes only children with a prenatal substantiation and in postnatal reports section includes children with only postnatal reports and substantiations. Suppression used to address privacy requirements for small cell sizes. NT aggregated with ACT, SA and Tas for prenatal reports to address privacy requirements due to small number of reports.

#### Children with Only Postnatal Reports

Aboriginal children form a larger percentage of prenatally reported children in both WA and Qld (47.9% and 40.2%) than children first reported postnatally (33.2% and 33.9% respectively) as shown in Table 1. Almost half of the children first reported postnatally had a single report (47.6%), with 42.1% having 2–5 reports and 10.3% having 6 or more reports. Emotional abuse was the most common maltreatment type recorded in first substantiations of post-natal reports (53.4%) followed by neglect (21.9%) and physical abuse (19.6%).

### Characteristics Associated with Prenatal Reports

Bivariate logistic regressions showed children in WA (OR 0.70, 95% CI: 0.65–0.75) and combined ACT, SA, Tas and NT (OR 0.21, 95% CI: 0.19–0.22) were significantly less likely than QLD children to be reported prenatally. Aboriginal children had almost doubled odds of having a prenatal report (OR 1.86, 95% CI: 1.77–1.95) than non-Aboriginal children.

### Timing of Children’s First Reports

**Figure 1.**
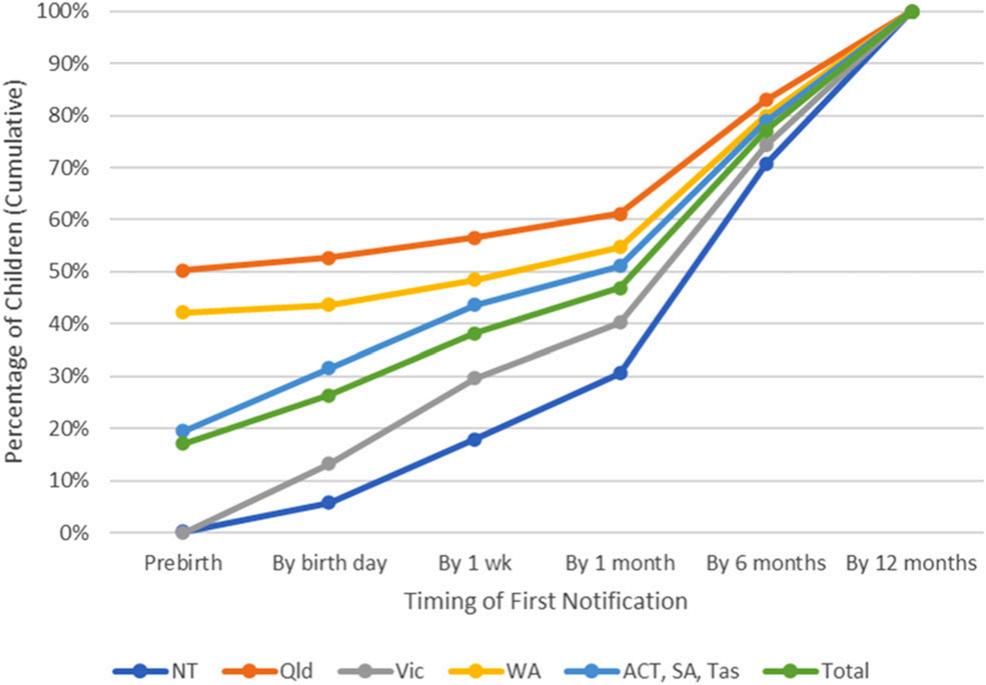
Timing of Children’s First Reports (Cumulative) by Jurisdiction

Half the study children’s first reports in Qld and 42% in WA occurred prenatally (Figure 1). In these jurisdictions, only 1%–2% were reported on the day of birth, compared to 6% in NT, 12% in the aggregated jurisdictions, and 15% in Vic. In NT (69%) and Vic (60%) (where prenatal reports are not recorded for the purpose of a child protection investigation), most children were first reported after 1 month.

### Entry to Out-of-Home Care

**Figure 2.**
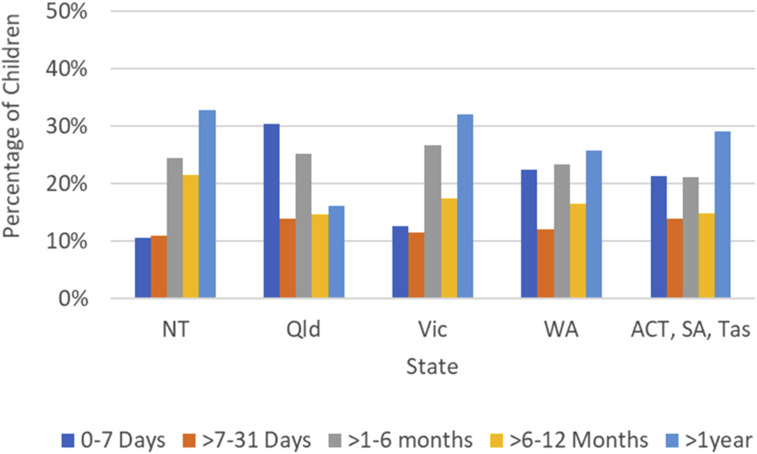
Timing of Children’s First Entry to OoHC, by Jurisdiction

In total, 18.5% of the children had entered care by the end of the study period (31st August 2018). The timing of children’s first entry to care varied by state. Newborn entries were most common in Queensland, and later entries, particularly beyond 1 year old were more common in Victoria and NT (Figure 2).

**Figure 3.**
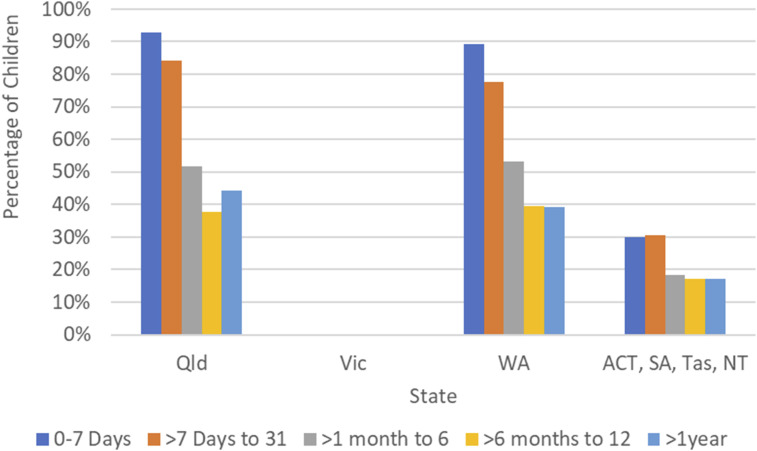
Timing of Children’s First Entry to OoHC and Percentage with a Prenatal Report, by Jurisdiction

In both Queensland (92.8%) and WA (89.2%), the majority of children who entered care in the first week after birth were those with a prenatal report (Figure 3). In these jurisdictions, over three quarters of children who had their first entry to care between 1 week and 1 month after birth were also subjects of a prenatal report. By comparison, just over half of the children who first entered care between one and 6 months old were the subject of a prenatal report.

### Jurisdictional Variation in Children Entering OoHC Accounting for Follow Up Time

As there may be differences in follow-up time (e.g., from Queensland’s later entry to the study) we calculated both the percentage of children who entered care by 1 and 2 years of age, excluding children without this duration of follow up (i.e., born near the end of the study period). Among children with at least 1 year follow up, 19.2% entered OoHC by age one, and with 2 years follow up, 20.3% entered by age two. In NT 12.3% entered OoHC by age two, 17.0% in ACT, SA and Tas combined, 21.4% in Vic, 23.0% in WA and 24.8% in Qld.

### Time from Birth to First Entry to OoHC by 1 Year Old

Results of regression analyses examining time to first OoHC are presented in Table 2.

**Table 2.** Survival Analysis of Time to OoHC by 1 Year Old

		Unadjusted estimates			
		Hazard ratio	95% CI		*p*
Sex	Male	(reference)			
	Female	0.97	0.93	1.02	.235
Aboriginal and Torres Strait Islander	No	(reference)			
	Yes	1.77	1.68	1.86	<.001
Jurisdiction	ACT, SA, Tas	(reference)			
	NT	0.66	0.57	0.76	<.001
	Qld	1.76	1.63	1.91	<.001
	Vic	1.22	1.14	1.31	<.001
	WA	1.46	1.34	1.58	<.001
Prenatal report	No prenatal reports	(reference)			
	Prenatal report	2.29	2.17	2.41	<.001
Time from first report to birth	No prenatal reports	(reference)			
	6–9 months before birth	2.30	2.05	2.58	<.001
	3–6 months before birth	2.59	2.41	2.79	<.001
	1–3 months before birth	2.02	1.84	2.22	<.001
	Final month before birth	1.67	1.46	1.92	<.001
Number of prenatal reports	No prenatal reports	(reference)			
	1	1.87	1.77	1.99	<.001
	2–5	2.51	2.26	2.80	<.001
	6+	5.76	3.72	8.94	<.001
Number of postnatal reports	No postnatal reports	(reference)			
	1	0.74	0.68	0.79	<.001
	2–5	0.52	0.48	0.56	<.001
	6+	0.39	0.35	0.44	<.001
Total number of reports	1	(reference)			
	2–5	0.83	0.79	0.87	<.001
	6+	0.60	0.54	0.66	<.001
Any prenatal substantiation	No prenatal substantiations	(reference)			
	Any prenatal substantiation	6.42	6.05	6.81	<.001
First substantiation type (prenatal)	Physical abuse	(reference)			
	Sexual abuse	0.91	0.59	1.42	.688
	Emotional abuse	0.72	0.59	0.88	.001
	Neglect	1.36	1.18	1.58	<.001

#### Timing of First Report

Having a prenatal report was associated with a 129% increased likelihood of entering OoHC (HR = 2.29, 95% CI: 2.17–2.41) compared to children with only postnatal reports amongst the whole study population (Table 2). Sensitivity analyses for time to OoHC were conducted including controlling for Aboriginal status and using follow-up data up to 6 years instead of 12 months, to ensure results did not simply reflect cross-jurisdictional variations in Aboriginal population or short-term variation in outcomes. Sensitivity analyses results were consistent with the findings presented here. In the states with prenatal reporting this was higher (QLD HR = 2.83, 95% CI: 2.51–3.19; WA HR = 3.01, 95% CI: 2.66–3.42), as shown in Supplemental Table 1. The increased likelihood associated with having a prenatal report compared to only postnatal reports was similar for Aboriginal (HR = 2.22, 95% CI: 2.04–2.41) and non-Aboriginal children (HR = 2.08, 95% CI: 1.93–2.23), as shown in Supplemental Table 2.

Time-stratified cox regression showed the increased likelihood of OoHC associated with prenatal reports was highest in the week after birth (HR: 4.65, 95% CI: 4.24–5.11), reducing over time (Supplemental Table 3). The relationship between timing during pregnancy of prenatal reports and entry to OoHC is shown in Table 2. Early pregnancy reports were not associated with reduced likelihood of OoHC. Compared to children not reported prenatally, the highest likelihood of OoHC was for children first reported three to six months before birth (HR: 2.59, 95% CI: 2.41–2.79), with lower likelihood for children reported in the month before birth (HR: 1.67, 95% CI: 1.46–1.92) (Table 2).

#### Child Demographics

Overall, children’s Aboriginal status was associated with a 77% increased likelihood of entering OoHC (HR: 1.77, 95% CI: 1.68–1.86) (Table 2). This varied greatly across jurisdictions. In NT, Aboriginal status was associated with a 361% increased likelihood of OoHC (HR: 4.61, 95% CI: 2.52 –8.44), 142% in Vic (HR = 2.42, 95% CI: 2.22–2.64), 123% in WA (HR: 2.23, 95% CI: 1.98–2.52), 101% in combined ACT, SA and Tas (HR: 2.01, 95% CI: 1.78–2.26), and 34% in Qld (HR: 1.34, 95% CI: 1.20–1.50) (Supplemental Table 1).

#### Jurisdiction

Compared to children in ACT, SA or Tas (combined), likelihood of OoHC was higher for children in Qld (HR: 1.76, 95% CI: 1.63–1.91), WA (HR: 1.46, 95% CI: 1.34–1.58) or Vic (HR: 1.22, 95% CI: 1.14–1.31) but lower in NT (HR: 0.66, 95% CI: 0.57–0.76). To assess whether jurisdictional variations in OoHC reflected more entries or simply earlier entries, logistic regression analysis of OoHC entry by jurisdiction was conducted. The logistic regression results (Supplemental Table 4) were consistent with the Cox regression results.

#### Reports and Substantiations

Compared to no prenatal reports (postnatal only), children with 1 prenatal report had almost doubled likelihood of entering OoHC (HR: 1.87, 1.77–1.99). The likelihood increased to HR: 5.76 (95% CI: 3.72–8.94) for over 5 prenatal reports. Conversely, higher numbers of total reports (and postnatal reports) had an inverse relationship with likelihood of OoHC (see also Supplemental Table 5).

Prenatal substantiations were associated with higher likelihood of OoHC (HR: 6.42, 95% CI: 6.05–6.81). The relationship varied by maltreatment type. Compared to children with a first prenatal substantiation of physical abuse, likelihood of OoHC entry was lower for emotional abuse (HR: 0.72, 95% CI: 0.59–0.88), and higher for neglect (HR: 1.36, 95% CI: 1.18–1.58).

### Reunifications

Overall, 21.8% of children experienced at least one reunification, varying from just over 14% in WA and ACT, SA and Tas, to 27.4% in NT and 34.7% in Vic. Most reunified children had only one reunification event during the study (to August 2018) (92.1%), 6.9% had two, and 0.9% three. The percentage of children who had more than one reunification events varied from 4.8% in ACT, SA, and Tas (combined), to 8.8% in Vic. Reunification figures may be different with longer follow-up. Cox regression analysis showed a significantly lower likelihood of reunification was found for prenatally reported children (HR: 0.41, 95% CI: 0.35–0.49), and Aboriginal children (HR: 0.72, 95% CI: 0.66–0.80) among others (Table 3).

**Table 3.** Survival Analysis Time From OoHC to Reunification

		Hazard Ratio	95% CI		*p*
Sex	Male	(reference)			
	Female	0.99	0.90	1.08	.749
Aboriginal and Torres Strait Islander status	Non-Aboriginal and Torres Strait Islander	(reference)			
	Aboriginal and Torres Strait Islander	0.73	0.66	0.80	<.001
Jurisdiction	ACT, SA, Tas	(reference)			
	NT	1.86	1.46	2.37	<.001
	Vic	2.68	2.34	3.07	<.001
	WA	0.91	0.75	1.10	.332
Prenatal report	No prenatal reports	(reference)			
	Prenatal report	0.41	0.35	0.49	<.001
Time from first report to birth	No prenatal reports	(reference)			
	6–9 months before birth	0.42	0.28	0.63	<.001
	3–6 months before birth	0.40	0.31	0.50	<.001
	1–3 months before birth	0.42	0.31	0.56	<.001
	Final month before birth	0.46	0.31	0.69	<.001
Number of prenatal reports	No prenatal reports	(reference)			
	1	0.42	0.35	0.51	<.001
	2–5	0.38	0.27	0.52	<.001
	6+	0.49	0.18	1.30	.149
Number of postnatal reports	No postnatal reports	(reference)			
	1	2.86	2.05	3.99	<.001
	2–5	4.03	2.90	5.60	<.001
	6+	4.39	3.13	6.15	<.001
Total number of reports	1	(reference)			
	2–5	1.42	1.29	1.58	<.001
	6+	1.51	1.33	1.72	<.001
Any prenatal substantiation	No prenatal substantiations				
	Any prenatal substantiation	0.40	0.32	0.49	<.001

## Discussion

This study used cross-jurisdictional data to examine the child protection pathways of children with prenatal and infant reports. We found the child protection pathways of infants reported prenatally differed significantly from those first reported postnatally as infants. Marked differences in children’s pathways across jurisdictions and for Aboriginal children were documented.

In the two jurisdictions with prenatal reporting (and not aggregated), around half the cases were reported prenatally (WA-42.9%, Qld-51.8%). Australia-wide, children with prenatal reports and substantiations were more likely to enter OoHC (overall and earlier) compared to children only reported postnatally. Higher numbers of prenatal reports were associated with increased likelihood of OoHC, consistent with previous research (Taplin, 2017). The negative relationship between higher numbers of postnatal/total reports and OoHC suggests children who enter OoHC early do not remain at home long enough to accumulate reports.

The types of maltreatment in first-substantiated prenatal and postnatal reports were somewhat similar, with neglect comprising the majority of prenatal reports, followed by emotional and physical abuse, and emotional abuse comprising the majority of post-natal reports followed by neglect and physical abuse. Note that emotional abuse includes exposure to domestic violence. As substantiation includes either harm or likelihood of harm, prenatal substantiations of sexual abuse are likely to reflect factors being considered such as a history of sexual offences or abuse of the unborn child’s older siblings.

We found over two-thirds (68.8%) of prenatal reports occurred in the second trimester of pregnancy or earlier, whereas a previous study found under half occurred before the third trimester (Taplin, 2017), possibly indicating a trend towards earlier reporting as systems become more established. Reporting earlier in pregnancy allows time for pre-birth planning and provision of services to reduce the need for removals. However, early reports had a higher likelihood of OoHC than late pregnancy reports. This raises questions regarding pre-birth processes and the effectiveness of planning and support. It is possible that an increased likelihood of OOHC entry among children reported earlier in pregnancy reflects the severity of issues in these families, which may reduce the effectiveness of planning and interventions. Families with perinatal reports often have multiple and complex needs including domestic violence, substance use, mental health concerns, intergenerational maltreatment (Meiksans et al., 2021), and housing/homelessness issues (Trew et al., 2022). Further research is needed to examine family characteristics associated with timing of prenatal reports to inform targeted support. Clearly, examining effectiveness and potential improvements in interventions during pregnancy could improve the likelihood of children remaining safely at home after birth.

Consistent across all levels of child protection systems, Aboriginal and Torres Strait Islander children were more likely to have prenatal reports, more likely to enter OoHC and less likely to be reunified compared to children who were not identified as Aboriginal or Torres Strait Islander. The disparity concerning OoHC entry was most evident in NT, where the proportional population of Aboriginal people is highest (Australian Bureau of Statistics, 2021b). It is recognised within Australia that the overrepresentation of Aboriginal children in the child protection system must be understood within a context that includes colonization and past policies of widespread forced child removals known as the ‘Stolen Generations’ resulting in separation from family and culture, and ongoing intergenerational trauma (Newton, 2020). Addressing this overrepresentation is considered a priority for the Australian Government (Commonwealth of Australia, 2021). As outlined by Chamberlain et al. (2022) Aboriginal-led strategies are needed, along with increased investment in prevention, early intervention and systemic transformation. This would include provision of culturally responsive, trauma-informed maternity and family-support services, and child protection delegated authority to Aboriginal services to best utilise the perinatal period for earlier intervention and support (Chamberlain et al., 2022).

There were marked differences in children’s child protection trajectories across jurisdictions. It is useful to consider this research alongside population rates of child protection involvement. Although the proportion of perinatally reported children entering OoHC was lowest in NT and ACT, SA, and Tas (combined), previous research shows these four jurisdictions combined had the highest population rates of infant OoHC and reports (O’Donnell et al., 2023). Together these findings highlight that high OoHC rates in this instance reflect differences in early child protection processes e.g. rates of reporting. Conversely, Vic was previously found to have the highest population rates of reports and OoHC for Aboriginal infants nationally (O’Donnell et al., 2023). Our study showed Vic was also the jurisdiction with the second highest increased likelihood of entering OoHC among Aboriginal infants reported to child protection. In this case, increased rates of OoHC reflect differences occurring at each step in the child protection pathway. Jurisdictions differed in the use of prenatal reporting, the likelihood of entering OoHC (with reported children in WA and Queensland most likely to enter OoHC), and likelihood of reunification (children in Vic and NT were twice as likely as other jurisdictions to have at least one reunification attempt). Further research is required to understand the nature and context of the prenatal reports across jurisdictions.

There are many potential drivers of this variability including population differences in risk factors and availability of government/non-government services, along with differences in jurisdictional policy frameworks. Our findings raise important questions - for jurisdictions without prenatal reporting, does the absence of prenatal reporting delay opportunities to support families to provide a protective home environment from birth?; for jurisdictions with prenatal reporting, why do infant removal rates remain high, what impact does (in)/voluntary parental engagement with child protection processes during pregnancy have, and could more be done during pregnancy to prevent infant removals? Further research is needed to examine the characteristics of prenatally-reported families in more detail; what interventions are currently provided, when and to which families; and the effectiveness of current and additional interventions for these families taking into account the characteristics and complex needs of the families involved.

### Limitations

Despite strengths including individual-level data covering multiple jurisdictions, and population data which reduces many forms of bias (e.g., sampling bias, attrition bias, social desirability bias), the study had limitations. Data was unavailable for NSW, the most populous Australian state. There may be differences in data recording practices, so some caution needs to be applied in comparing jurisdictions, however this has been addressed to some extent through standardised reporting requirements and analysis focussing on the number of children that experience child protection event rather than counting the number of events. No data was available on family factors (e.g., domestic violence, mental health, substance use, siblings already in OoHC), so the relationship of these with report timing and children’s child protection outcomes could not be examined. Although this could not be undertaken using the national data, we plan to examine family factors related to perinatal reporting using state data which includes these variables. Likewise, we did not have data on service provision to examine whether, when and how interventions were implemented. Further research examining family factors, policy differences, and service provision is recommended.

### Conclusion

Prenatal reporting is becoming more common. Rates of prenatal reporting increased by 4% per year in Australia between 2012–13 and 2018–19, with marked variation between jurisdictions: from a 3% annual increase in NSW to 15% in WA (O’Donnell et al., 2023). This study provides new information on prenatal and infant reporting, how it varies across jurisdictions, and the child protection pathways and outcomes of these children. Policy, practice and population differences interact to produce different outcomes and there is a need to better understand how each of these factors are contributing to the outcomes for infants reported perinatally and the marked jurisdictional variations. Research also needs to be conducted to understand when and what supports are provided to these families, how effective they are and what additional supports may be effective in ensuring a safe family environment. The findings from this study, in combination with more detailed research should be used to further our understanding of how best to intervene and support different populations of families to improve infant and family outcomes.

## Supplemental Material

Supplemental Material - Prenatal and Infant Reports and Child Protection Involvement: A Longitudinal Cohort Study Supplemental Material for Prenatal and Infant Reports and Child Protection Involvement: A Longitudinal Cohort Study by Miriam J. Maclean, Fernando Lima, Stephanie Taplin, Rhonda Marriott, Jacynta Krakouer, and Melissa O’Donnell in Child Maltreatment

## Data Availability

The data that support the findings of this study are available from the Australian Institute of Health and Welfare but restrictions apply to the availability of these data, which were provided and approved for the current study, and so are not publicly available. Data may be available from the authors upon reasonable request and with permission of the Australian Institute of Health and Welfare and conditional upon complying with the requirements of the data governance framework.*

## Appendix

### Abbreviations

CI: Confidence Interval
HR: Hazard Ratio
OR: Odds Ratio
OoHC: Out-of-Home Care
AIHW: Australian Institute of Health and Welfare
ACT: Australian Capital Territory
NSW: New South Wales
NT: Northern Territory
Qld: Queensland
SA: South Australia
Tas: Tasmania
WA: Western Australia
Vic: Victoria.

## References

[bibr1-10775595251381279] Amos J. Todd B. Gibson B. Carpenter S. Malvaso C. G. Delfabbro P. H. (2022). Using the adult exploration of attachment interview (AEAI) to break the cycle of intergenerational trauma: Illustrations from a family reunification program. Australian and New Zealand Journal of Family Therapy, 43(2), 168–181. 10.1002/anzf.1490

[bibr3-10775595251381279] Australian Bureau of Statistics . (2021a). Census all persons QuickStats. Retrieved 08 June 2023 from. https://www.abs.gov.au/census/find-census-data/search-by-area

[bibr4-10775595251381279] Australian Bureau of Statistics . (2021b). Aboriginal and Torres Strait Islander people: Census [internet]. Retrieved 27 April 2023 from. https://www.abs.gov.au/statistics/people/aboriginal-and-torres-strait-islander-peoples/aboriginal-and-torres-strait-islander-people-census/2021

[bibr2-10775595251381279] Australian Institute of Health and Welfare . (2022). Child protection Australia 2020–21. AIHW, Australian Government. https://www.aihw.gov.au/getmedia/2cecbc62-8c65-4031-a6df-5fec9422b0cf/child-protection-australia-2021-22.pdf?v=20240828142750&inline=true

[bibr5-10775595251381279] Blythe S. Elcombe E. Peters K. Burns E. Gribble K. (2022). Australian foster carers’ views of supporting maternal breastfeeding and attachment in out-of-home care. Child Abuse & Neglect, 130(Pt 3), 105360. 10.1016/j.chiabu.2021.105360 34688491

[bibr6-10775595251381279] Broadhurst K. Mason C. Ward H. (2022). Urgent care proceedings for new-born babies in England and Wales – time for a fundamental review. International Journal of Law, Policy and the Family, 36(1), ebac008. 10.1093/lawfam/ebac008

[bibr7-10775595251381279] Brownell M. D. Chartier M. Au W. MacWilliam L. Schultz J. Guenette W. Valdivia J. (2015). The educational outcomes of children in care in Manitoba.

[bibr8-10775595251381279] Chamberlain C. Gray P. Bennet D. Elliott A. Jackomos M. Krakouer J. Marriott R. O’Dea B. Andrews J. Andrews S. Atkinson C. Atkinson J. Bhathal A. Bundle G. Davies S. Herrman H. Hunter S. A. Jones‐Terare G. Leane C. Langton M. (2022). Supporting Aboriginal and Torres Strait Islander families to stay together from the start (SAFeST start): Urgent call to action to address crisis in infant removals. The Australian Journal of Social Issues, 57(2), 252–273. 10.1002/ajs4.200 35910416 PMC9304314

[bibr9-10775595251381279] Cicchetti D. (2016). Socioemotional, personality, and biological development: Illustrations from a multilevel developmental psychopathology perspective on child maltreatment. Annual Review of Psychology, 67(1), 187–211. 10.1146/annurev-psych-122414-033259 26726964

[bibr10-10775595251381279] Commonwealth of Australia . (2020). National Agreement on Closing the Gap. https://www.closingthegap.gov.au/national-agreement/national-agreement-closing-the-gap

[bibr11-10775595251381279] Commonwealth of Australia . (2021). Safe and supported: The national framework for protecting Australia’s children 2021–2031. Commonwealth of Australia (Department of Social Services). https://www.dss.gov.au/system/files/resources/dess5016-national-framework-protecting-childrenaccessible.pdf

[bibr12-10775595251381279] Eastman A. L. Mitchell M. N. Putnam-Hornstein E. (2016). Risk of re-report: A latent class analysis of infants reported for maltreatment. Child Abuse & Neglect, 55, 22–31. 10.1016/j.chiabu.2016.03.002 27082751

[bibr13-10775595251381279] Gilbert R. Fluke J. O'Donnell M. Gonzalez-Izquierdo A. Brownell M. Gulliver P. Janson S. Sidebotham P. (2012). Child maltreatment: Variation in trends and policies in six developed countries. Lancet. https://linkinghub.elsevier.com/retrieve/pii/S0140673611610878 10.1016/S0140-6736(11)61087-8 22169108

[bibr14-10775595251381279] Juhasz I. B. (2020). Child welfare and future assessments–An analysis of discretionary decision-making in newborn removals in Norway. Children and Youth Services Review, 116, 105137. 10.1016/j.childyouth.2020.105137

[bibr15-10775595251381279] Marsh C. A. Browne J. Taylor J. Davis D. (2017). Characteristics and outcomes of newborns entered who entered into care (EIC) within 7 days of birth in NSW, Australia. Children and Youth Services Review, 81, 261–267. 10.1016/j.childyouth.2017.08.005

[bibr16-10775595251381279] Meiksans J. Arney F. Flaherty R. Octoman O. Chong A. Ward F. Taylor C. (2021). Risk factors identified in prenatal child protection reports. Children and Youth Services Review, 122, 105905. 10.1016/j.childyouth.2020.105905

[bibr17-10775595251381279] Newton B. (2020). Aboriginal parents’ experiences of having their children removed by statutory child protection services. Child & Family Social Work, 25(4), 814–822. 10.1111/cfs.12759

[bibr18-10775595251381279] O’Donnell M. Lima F. Maclean M. Marriott R. Taplin S. (2023). Infant and pre-birth involvement with child protection across Australia. Child Maltreatment, 28(4), 608–620. 10.1177/10775595231186647 37386757 PMC10540487

[bibr19-10775595251381279] O’Donnell M. Taplin S. Marriott R. Lima F. Stanley F. J. (2019). Infant removals: The need to address the over-representation of Aboriginal infants and community concerns of another ‘stolen generation’. Child Abuse & Neglect, 90, 88–98. 10.1016/j.chiabu.2019.01.017 30769191

[bibr20-10775595251381279] Office of the Children’s Commissioner . (2020). Te Kuku O Te Manawa – Ka puta te riri, ka momori te ngākau, ka heke ngā roimata mo tōku pēpi. Report 1. New Zealand. https://www.manamokopuna.org.nz/publications/reports/te-kuku-o-te-manawa/

[bibr21-10775595251381279] Pearson R. J. Jay M. A. O’Donnell M. Wijlaars L. Gilbert R. (2020). Characterizing newborn and older infant entries into care in England between 2006 and 2014. Child Abuse & Neglect, 109, 104760. 10.1016/j.chiabu.2020.104760 33053479 PMC7718112

[bibr22-10775595251381279] Swain S. (2014). History of child protection legislation. Royal Commission into Institutional Responses to Child Sexual Abuse.

[bibr23-10775595251381279] Taplin S. (2017). Prenatal reporting to child protection: Characteristics and service responses in one Australian jurisdiction. Child Abuse & Neglect, 65, 68–76. 10.1016/j.chiabu.2017.01.007 28113086

[bibr24-10775595251381279] Trew S. Taplin S. O’Donnell M. Marriott R. Broadhurst K. (2022). Parents’ experiences with child protection during pregnancy and post‐birth. Child and Family Social Work, 28(2). 10.1111/cfs.12984

[bibr25-10775595251381279] Victorian Department of Families Fairness and Housing . (2023). Child protection manual. https://www.cpmanual.vic.gov.au/policies-and-procedures/phases/intake/unborn-child-reports

