# Supplementary material for: Prenatal and Infant Reports and Child Protection Involvement: A Longitudinal Cohort Study
Source: Child Maltreat. 2025 Sep 19;31(3):479–89. doi: 10.1177/10775595251381279 (PMC13264657; doi:10.1177/10775595251381279)

Supplemental Table 1. Survival Analysis of Time to OoHC Stratified by Jurisdiction

| | | Hazard ratio | 95% CIs | | p |
|---|---|---|---|---|---|
| Aboriginal and Torres Strait Islander | | | | | |
| NT | No | (reference) | | | |
| | Yes | 4.61 | 2.52 | 8.44 | <.001 |
| Qld | No | (reference) | | | |
| | Yes | 1.34 | 1.20 | 1.50 | <.001 |
| Vic | No | (reference) | | | |
| | Yes | 2.42 | 2.22 | 2.64 | <.001 |
| WA | No | (reference) | | | |
| | Yes | 2.23 | 1.98 | 2.52 | <.001 |
| ACT, SA, Tas | No | (reference) | | | |
| | Yes | 2.01 | 1.78 | 2.26 | <.001 |
| Prenatal report | | | | | |
| Qld | No prenatal reports | (reference) | | | |
| | Prenatal report | 2.83 | 2.51 | 3.19 | <.001 |
| WA | No prenatal reports | (reference) | | | |
| | Prenatal report | 3.01 | 2.66 | 3.42 | <.001 |
| ACT, SA, Tas | No prenatal reports | (reference) | | | |
| | Prenatal report | 1.83 | 1.61 | 2.07 | <.001 |
| Number of prenatal reports | | | | | |
| Qld | No prenatal reports | (reference) | | | |
| | 1 | 2.31 | 2.04 | 2.62 | <.001 |
| | 2-5 | 3.36 | 2.77 | 4.06 | <.001 |
| WA | No prenatal reports | (reference) | | | |
| | 1 | 2.84 | 2.50 | 3.23 | <.001 |
| | 2-5 | 2.94 | 2.18 | 3.96 | <.001 |
| ACT, SA, Tas | No prenatal reports | (reference) | | | |
| | 1 | 0.78 | 0.65 | 0.93 | .006 |
| | 2-5 | 2.42 | 2.05 | 2.84 | <.001 |
| | 6+ | 6.38 | 4.10 | 9.95 | <.001 |
| Number of postnatal reports | | | | | |
| NT | 1 | (reference) | | | |
| | 2-5 | 2.14 | 1.50 | 3.06 | <.001 |
| | 6+ | 2.52 | 1.71 | 3.71 | <.001 |
| Qld | No postnatal reports | (reference) | | | |
| | 1 | 0.61 | 0.54 | 0.69 | <.001 |
| | 2-5 | 0.59 | 0.51 | 0.69 | <.001 |
| | 6+ | 1.08 | 0.40 | 2.88 | .885 |
| Vic | 1 | (reference) | | | |
| | 2-5 | 0.54 | 0.50 | 0.59 | <.001 |
| | 6+ | 0.35 | 0.30 | 0.42 | <.001 |
| WA | No postnatal reports | (reference) | | | |

| | | | | | |
|---|---|---|---|---|---|
| | 1 | 0.51 | 0.45 | 0.59 | <.001 |
| | 2-5 | 0.50 | 0.43 | 0.58 | <.001 |
| | 6+ | 0.21 | 0.09 | 0.47 | <.001 |
| ACT, SA, Tas | No postnatal reports | (reference) | | | |
| | 1 | 2.19 | 1.71 | 2.82 | <.001 |
| | 2-5 | 1.99 | 1.55 | 2.55 | <.001 |
| | 6+ | 1.73 | 1.31 | 2.28 | <.001 |
| Total number of reports | | | | | |
| NT | 1 | (reference) | | | |
| | 2-5 | 2.14 | 1.49 | 3.05 | <.001 |
| | 6+ | 2.51 | 1.71 | 3.70 | <.001 |
| Qld | 1 | (reference) | | | |
| | 2-5 | 1.37 | 1.22 | 1.54 | <.001 |
| | 6+ | 0.74 | 0.31 | 1.78 | .502 |
| Vic | 1 | (reference) | | | |
| | 2-5 | 0.54 | 0.50 | 0.59 | <.001 |
| | 6+ | 0.35 | 0.30 | 0.42 | <.001 |
| WA | 1 | (reference) | | | |
| | 2-5 | 1.11 | 0.98 | 1.25 | .094 |
| | 6+ | 0.46 | 0.25 | 0.86 | .015 |
| ACT, SA, Tas | 1 | (reference) | | | |
| | 2-5 | 1.24 | 1.09 | 1.41 | .001 |
| | 6+ | 1.28 | 1.09 | 1.51 | .003 |
| Any prenatal substantiation | | | | | |
| Qld | No prenatal substantiations | (reference) | | | |
| | Any prenatal substantiation | 7.05 | 6.29 | 7.90 | <.001 |
| | No prenatal substantiations | (reference) | | | |
| WA | Any prenatal substantiation | 8.84 | 7.83 | 9.98 | <.001 |
| | No prenatal substantiations | (reference) | | | |
| ACT, SA, Tas | Any prenatal substantiation | 6.32 | 5.26 | 7.60 | <.001 |
| First substantiation type (prenatal) | | | | | |
| Qld | Physical abuse | (reference) | | | |
| | Sexual abuse | 1.05 | 0.64 | 1.73 | .837 |
| | Emotional abuse | 0.77 | 0.57 | 1.05 | .101 |
| | Neglect | 1.11 | 0.92 | 1.35 | .274 |
| WA | Physical abuse | (reference) | | | |
| | Sexual abuse | 0.51 | 0.16 | 1.60 | .245 |
| | Emotional abuse | 0.72 | 0.54 | 0.96 | .025 |
| | Neglect | 1.76 | 1.40 | 2.21 | <.001 |
| ACT, SA, Tas | Physical abuse | (reference) | | | |
| | Sexual abuse | 0.95 | 0.11 | 8.17 | .966 |
| | Emotional abuse | 1.17 | 0.46 | 2.98 | .744 |
| | Neglect | 3.07 | 1.24 | 7.60 | .015 |

Supplemental Table 2. Survival Analysis Time to OoHC by Aboriginal and Torres Strait Islander Status

| | Aboriginal and Torres Strait Islander | | | | Non-Aboriginal and Torres Strait Islander | | | |
|---|---|---|---|---|---|---|---|---|
| | Hazard ratio | 95% CI | | p | Hazard ratio | 95% CI | | p |
| Sex | | | | | | | | |
| Male | (reference) | | | | | | | |
| Female | 0.93 | 0.86 | 1.01 | .078 | 1.00 | 0.94 | 1.07 | .941 |
| Prenatal report | | | | | | | | |
| No prenatal reports | | | | | | | | |
| Prenatal report | 2.22 | 2.04 | 2.41 | <.001 | 2.08 | 1.93 | 2.23 | <.001 |
| Jurisdiction | | | | | | | | |
| ACT, SA, Tas | (reference) | | | | | | | |
| NT | 0.46 | 0.39 | 0.54 | <.001 | 0.2 | 0.11 | 0.37 | <.001 |
| Qld | 1.26 | 1.11 | 1.43 | <.001 | 1.89 | 1.71 | 2.09 | <.001 |
| Vic | 1.52 | 1.35 | 1.73 | <.001 | 1.28 | 1.18 | 1.39 | <.001 |
| WA | 1.33 | 1.17 | 1.51 | <.001 | 1.2 | 1.07 | 1.34 | .002 |
| Time from first report to birth | | | | | | | | |
| No prenatal reports | (reference) | | | | | | | |
| 6 - 9 months before birth | 2.54 | 2.15 | 3.00 | <.001 | 1.95 | 1.66 | 2.28 | <.001 |
| 3-6 months before birth | 2.36 | 2.12 | 2.63 | <.001 | 2.43 | 2.2 | 2.69 | <.001 |
| 1-3 months before birth | 2.01 | 1.75 | 2.3 | <.001 | 1.8 | 1.58 | 2.05 | <.001 |
| Final month before birth | 1.58 | 1.29 | 1.95 | <.001 | 1.59 | 1.33 | 1.91 | <.001 |
| Number of prenatal reports | | | | | | | | |
| No prenatal reports | (reference) | | | | | | | |
| 1 | 1.97 | 1.81 | 2.15 | <.001 | 1.59 | 1.47 | 1.73 | <.001 |
| 2-5 | 2.35 | 1.98 | 2.78 | <.001 | 2.46 | 2.15 | 2.82 | <.001 |
| 6+ | 6.36 | 3.03 | 13.37 | <.001 | 5.5 | 3.19 | 9.48 | <.001 |
| Number of postnatal reports | | | | | | | | |
| Postnatal reports 0 | (reference) | | | | | | | |
| 1 | 0.74 | 0.66 | 0.82 | <.001 | 0.86 | 0.78 | 0.94 | <.001 |
| 2-5 | 0.41 | 0.37 | 0.46 | <.001 | 0.64 | 0.58 | 0.71 | <.001 |
| 6+ | 0.31 | 0.26 | 0.37 | <.001 | 0.45 | 0.38 | 0.53 | <.001 |
| Total number of reports | | | | | | | | |
| 1 | (reference) | | | <.001 | | | | |

| | | | | | | | | |
|---|---|---|---|---|---|---|---|---|
| 2-5 | 0.65 | 0.6 | 0.71 | <.001 | 0.87 | 0.82 | 0.93 | <.001 |
| 6+ | 0.44 | 0.38 | 0.52 | <.001 | 0.62 | 0.55 | 0.71 | <.001 |
| Any prenatal substantiation | | | | | | | | |
| No prenatal substantiations | (reference) | | | | | | | |
| Any prenatal substantiation | 4.93 | 4.52 | 5.39 | <.001 | 6.84 | 6.31 | 7.43 | <.001 |
| First substantiation type (prenatal) | (reference) | | | | | | | |
| Physical abuse | (reference) | | | | | | | |
| Sexual abuse | 0.86 | 0.42 | 1.76 | .687 | 0.96 | 0.55 | 1.68 | .890 |
| Emotional abuse | 0.74 | 0.57 | 0.97 | .029 | 0.71 | 0.53 | 0.94 | .016 |
| Neglect | 1.41 | 1.15 | 1.71 | .001 | 1.34 | 1.08 | 1.66 | .008 |

Supplemental Table 3. Survival Analysis Time to OoHC Stratified by Time

| | Time from birth to OOHC | | Hazard Ratio | 95% CIs | | p |
|---|---|---|---|---|---|---|
| Aboriginal and Torres Strait Islander | | | | | | |
| | 0-7 Days | No | (reference) | | | |
| | | Yes | 1.86 | 1.69 | 2.05 | <.001 |
| | 8-31 Days | No | (reference) | | | |
| | | Yes | 1.85 | 1.64 | 2.09 | <.001 |
| | 1-6 Months | No | (reference) | | | |
| | | Yes | 1.70 | 1.56 | 1.86 | <.001 |
| | 6-12 Months | No | (reference) | | | |
| | | Yes | 1.71 | 1.54 | 1.90 | <.001 |
| Prenatal report | | | | | | |
| | 0-7 Days | No prenatal reports | (reference) | | | |
| | | Prenatal report | 4.65 | 4.24 | 5.11 | <.001 |
| | 8-31 Days | No prenatal reports | (reference) | | | |
| | | Prenatal report | 2.83 | 2.50 | 3.22 | <.001 |
| | 1-6 Months | No prenatal reports | (reference) | | | |
| | | Prenatal report | 1.47 | 1.32 | 1.63 | <.001 |
| | 6-12 Months | No prenatal reports | (reference) | | | |
| | | Prenatal report | 1.19 | 1.03 | 1.36 | .015 |
| Jurisdiction | | | | | | |
| | 0-7 Days | ACT, SA, Tas | (reference) | | | |
| | | NT | 0.38 | 0.27 | 0.52 | <.001 |
| | | Qld | 1.93 | 1.68 | 2.21 | <.001 |
| | | Vic | 0.77 | 0.68 | 0.88 | <.001 |
| | | WA | 1.43 | 1.23 | 1.66 | <.001 |
| | 8-31 Days | ACT, SA, Tas | (reference) | | | |
| | | NT | 0.50 | 0.35 | 0.73 | <.001 |
| | | Qld | 1.48 | 1.22 | 1.79 | <.001 |
| | | Vic | 1.06 | 0.90 | 1.24 | .495 |
| | | WA | 1.18 | 0.96 | 1.45 | .110 |
| | 1-6 Months | ACT, SA, Tas | (reference) | | | |
| | | NT | 0.81 | 0.64 | 1.02 | .075 |
| | | Qld | 1.81 | 1.57 | 2.09 | <.001 |
| | | Vic | 1.59 | 1.41 | 1.80 | <.001 |
| | | WA | 1.53 | 1.31 | 1.77 | <.001 |
| | 6-12 Months | ACT, SA, Tas | (reference) | | | |
| | | NT | 1.00 | 0.77 | 1.30 | .994 |
| | | Qld | 1.64 | 1.37 | 1.97 | <.001 |
| | | Vic | 1.52 | 1.32 | 1.75 | <.001 |
| | | WA | 1.63 | 1.36 | 1.94 | <.001 |

| | | | | | |
|---|---|---|---|---|---|
| Time from first report to birth | | | | | |
| 0-7 Days | No prenatal reports | (reference) | | | |
| | 6 - 9 months before birth | 5.63 | 4.77 | 6.64 | <.001 |
| | 3-6 months before birth | 5.54 | 4.92 | 6.22 | <.001 |
| | 1-3 months before birth | 3.49 | 2.98 | 4.09 | <.001 |
| | Final month before birth | 2.81 | 2.22 | 3.55 | <.001 |
| 8-31 Days | No prenatal reports | (reference) | | | |
| | 6 - 9 months before birth | 1.97 | 1.45 | 2.69 | <.001 |
| | 3-6 months before birth | 3.40 | 2.88 | 4.01 | <.001 |
| | 1-3 months before birth | 2.75 | 2.24 | 3.37 | <.001 |
| | Final month before birth | 2.08 | 1.52 | 2.83 | <.001 |
| 1-6 Months | No prenatal reports | (reference) | | | |
| | 6 - 9 months before birth | 1.18 | 0.92 | 1.53 | .196 |
| | 3-6 months before birth | 1.43 | 1.23 | 1.67 | <.001 |
| | 1-3 months before birth | 1.60 | 1.35 | 1.90 | <.001 |
| | Final month before birth | 1.40 | 1.10 | 1.79 | .006 |
| 6-12 Months | No prenatal reports | (reference) | | | |
| | 6 - 9 months before birth | 1.36 | 1.01 | 1.83 | .044 |
| | 3-6 months before birth | 1.30 | 1.06 | 1.59 | .010 |
| | 1-3 months before birth | 0.98 | 0.75 | 1.27 | .866 |
| | Final month before birth | 0.94 | 0.66 | 1.35 | .750 |
| Number of prenatal reports | | | | | |
| 0-7 Days | No prenatal reports | (reference) | | | |
| | 1 | 3.93 | 3.56 | 4.34 | <.001 |
| | 2-5 | 5.01 | 4.23 | 5.94 | <.001 |
| | 6+ | 9.04 | 4.51 | 18.13 | <.001 |
| 8-31 Days | No prenatal reports | (reference) | | | |
| | 1 | 2.26 | 1.97 | 2.60 | <.001 |
| | 2-5 | 3.44 | 2.72 | 4.35 | <.001 |
| | 6+ | 13.91 | 6.93 | 27.93 | <.001 |
| 1-6 Months | No prenatal reports | (reference) | | | |
| | 1 | 1.21 | 1.08 | 1.36 | .001 |
| | 2-5 | 1.59 | 1.28 | 1.97 | <.001 |
| | 6+ | 1.75 | 0.44 | 7.01 | .428 |
| 6-12 Months | No prenatal reports | (reference) | | | |
| | 1 | 0.94 | 0.80 | 1.09 | .398 |
| | 2-5 | 1.32 | 1.00 | 1.76 | .051 |
| | 6+ | 2.66 | 0.66 | 10.63 | .168 |
| Number of postnatal reports | | | | | |
| 0-7 Days | No postnatal reports | (reference) | | | |
| | 1 | 0.36 | 0.33 | 0.40 | <.001 |
| | 2-5 | 0.12 | 0.10 | 0.14 | <.001 |
| | 6+ | 0.07 | 0.05 | 0.10 | <.001 |
| 8-31 Days | No postnatal reports | (reference) | | | |

| | | | | | | |
|---|---|---|---|---|---|---|
| | | 1 | 0.68 | 0.58 | 0.79 | <.001 |
| | | 2-5 | 0.27 | 0.22 | 0.32 | <.001 |
| | | 6+ | 0.11 | 0.07 | 0.17 | <.001 |
| | 1-6 Months | No postnatal reports | (reference) | | | |
| | | 1 | 1.72 | 1.46 | 2.04 | <.001 |
| | | 2-5 | 1.28 | 1.08 | 1.52 | .005 |
| | | 6+ | 0.84 | 0.67 | 1.06 | .145 |
| | 6-12 Months | No postnatal reports | (reference) | | | |
| | | 1 | 4.27 | 2.86 | 6.38 | <.001 |
| | | 2-5 | 6.85 | 4.60 | 10.20 | <.001 |
| | | 6+ | 6.65 | 4.39 | 10.08 | <.001 |
| Total number of reports | | | | | | |
| | 0-7 Days | 1 | (reference) | | | |
| | | 2-5 | 0.43 | 0.39 | 0.48 | <.001 |
| | | 6+ | 0.23 | 0.18 | 0.30 | <.001 |
| | 8-31 Days | 1 | (reference) | | | |
| | | 2-5 | 0.49 | 0.43 | 0.56 | <.001 |
| | | 6+ | 0.28 | 0.21 | 0.38 | <.001 |
| | 1-6 Months | 1 | (reference) | | | |
| | | 2-5 | 0.96 | 0.88 | 1.05 | .398 |
| | | 6+ | 0.60 | 0.51 | 0.72 | <.001 |
| | 6-12 Months | 1 | (reference) | | | |
| | | 2-5 | 2.10 | 1.87 | 2.36 | <.001 |
| | | 6+ | 2.00 | 1.69 | 2.36 | <.001 |
| Any prenatal substantiation | | | | | | |
| | 0-7 Days | No prenatal substantiations | (reference) | | | |
| | | Any prenatal substantiation | 12.53 | 11.39 | 13.79 | <.001 |
| | 8-31 Days | No prenatal substantiations | (reference) | | | |
| | | Any prenatal substantiation | 7.87 | 6.87 | 9.01 | <.001 |
| | 1-6 Months | No prenatal substantiations | (reference) | | | |
| | | Any prenatal substantiation | 3.92 | 3.47 | 4.43 | <.001 |
| | 6-12 Months | No prenatal substantiations | (reference) | | | |
| | | Any prenatal substantiation | 2.50 | 2.06 | 3.03 | <.001 |
| First substantiation type (prenatal) | | | | | | |
| | 0-7 Days | Physical abuse | (reference) | | | |
| | | Sexual abuse | 0.70 | 0.36 | 1.38 | .302 |
| | | Emotional abuse | 0.54 | 0.40 | 0.72 | <.001 |
| | | Neglect | 1.28 | 1.04 | 1.56 | .017 |
| | 8-31 Days | Physical abuse | (reference) | | | |
| | | Sexual abuse | 1.56 | 0.66 | 3.71 | .312 |
| | | Emotional abuse | 0.96 | 0.61 | 1.50 | .844 |
| | | Neglect | 1.83 | 1.29 | 2.62 | .001 |
| | 1-6 Months | Physical abuse | (reference) | | | |
| | | Sexual abuse | 0.71 | 0.26 | 1.96 | .507 |

| | | | | | |
|---|---|---|---|---|---|
| | Emotional abuse | 0.85 | 0.59 | 1.24 | .410 |
| | Neglect | 1.16 | 0.85 | 1.57 | .347 |
| 6-12 Months | Physical abuse | (reference) | | | |
| | Sexual abuse | 1.75 | 0.51 | 5.99 | .376 |
| | Emotional abuse | 1.06 | 0.55 | 2.04 | .866 |
| | Neglect | 1.60 | 0.93 | 2.74 | .092 |

Supplemental Table 4. Logistic Regression Entry to OoHC by 2 years old by Jurisdiction

| | Odds Ratio | 95% CIs | | P |
|---|---|---|---|---|
| ACT, SA, Tas | (reference) | | | |
| NT | 0.67 | 0.57 | 0.78 | <.001 |
| Qld | 1.78 | 1.60 | 1.97 | <.001 |
| Vic | 1.27 | 1.18 | 1.37 | <.001 |
| WA | 1.50 | 1.36 | 1.66 | <.001 |

Note: Children with less than two years follow up time are excluded

Supplemental Table 5. Age at First OoHC Entry by Total Number of Reports

| | Total Number of Reports | | | | | |
|---|---|---|---|---|---|---|
| Age at first OoHC | 1 | | 2-5 | | 6+ | |
| 0-7days | 1,210 | 33.0% | 448 | 10.4% | 56 | 4.3% |
| > 7 days - 1 month | 770 | 21.0% | 331 | 7.7% | 44 | 3.4% |
| >1 month to 6 months | 1,138 | 31.0% | 1,010 | 23.5% | 152 | 11.6% |
| >6 months to 12 months | 423 | 11.5% | 891 | 20.8% | 209 | 16.0% |
| >1year | 128 | 3.5% | 1,610 | 37.5% | 846 | 64.7% |
| Total | 3,669 | 100.0% | 4,290 | 100.0% | 1,307 | 100.0% |

*Supplemental Figure 1. Time to First OoHC Entry by Jurisdiction, Extended Follow-up to 6 Years*

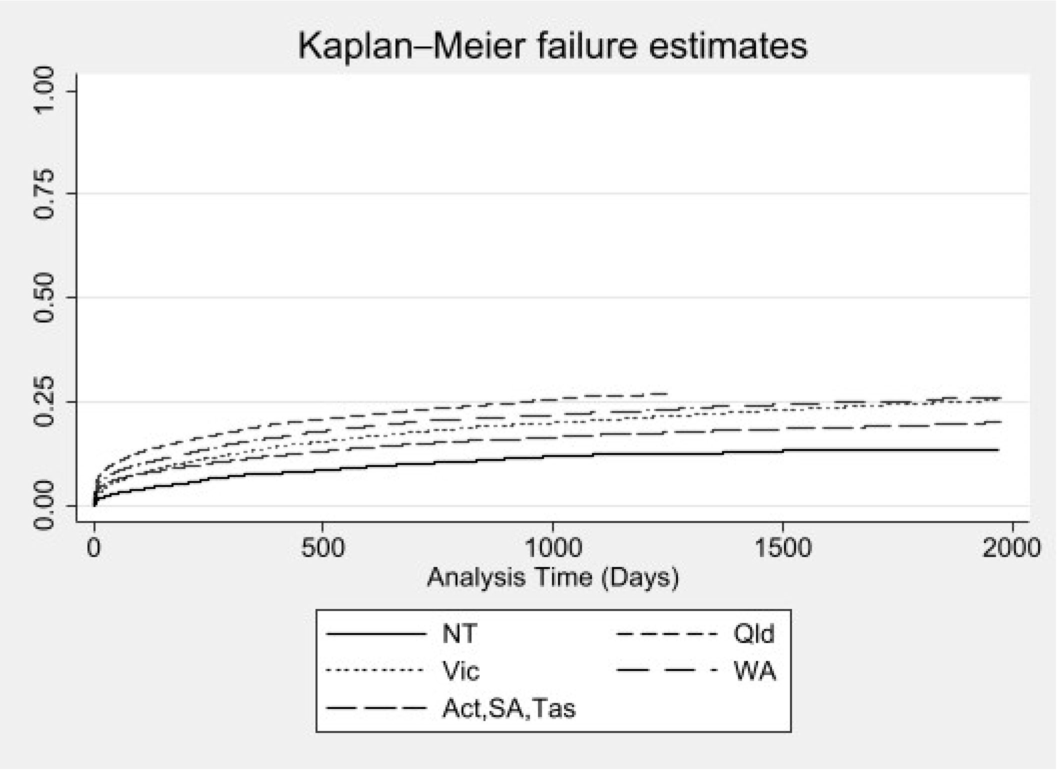

Supplement: Supplemental Material - Prenatal and Infant Reports and Child Protection Involvement: A Longitudinal Cohort Study [file sj-pdf-1-cmx-10.1177_10775595251381279.pdf]
